# A Community-Based Survivorship Model for Individuals With Orthopedic Disabilities in Rural Areas

**DOI:** 10.7759/cureus.87887

**Published:** 2025-07-14

**Authors:** Radhika A Jadhav, Sandeep Shinde, Shamika S Baraskar, Harshal Y Kale

**Affiliations:** 1 Musculoskeletal Sciences, Krishna Vishwa Vidyapeeth (Deemed to Be University), Karad, IND; 2 Musculoskeletal Sciences, Krishna College of Physiotherapy, Krishna Vishwa Vidyapeeth (Deemed to Be University), Karad, IND; 3 Critical Care Medicine, Krishna Vishwa Vidyapeeth (Deemed to Be University), Karad, IND

**Keywords:** community, disability, model, rural areas, survivorship

## Abstract

Background: Orthopedic disabilities are a significant public health challenge in rural areas, where access to rehabilitation and support services is often inadequate. Individuals with physical disabilities frequently lack the necessary awareness, resources, and community support to manage their conditions effectively.

Objective: This comparative study aims to evaluate a community-based survivorship model (CBSM) for individuals with orthopedic disabilities in rural areas. The model focuses on educating patients, fostering positive health behaviors, and providing a structured framework for managing disability-related challenges.

Methods: A study was conducted using primary data. A total of 156 participants meeting inclusion criteria were randomly allocated to intervention (n = 78) and control (n = 78) groups. The intervention group received the CBSM for three months, comprising six components: rehabilitation, prosthetics/orthotics, mental health support, health campaigns, occupational therapy, and financial aid. Outcomes were assessed pre- and post-intervention using the Visual Analogue Scale (VAS), Functional Independence Measure (FIM), Self-Efficacy Scale, and Patient Health Questionnaire-9 (PHQ-9). Data were analysed using IBM SPSS Statistics for Windows, Version 26 (Released 2020; IBM Corp., Armonk, New York, United States).

Results: The experimental group demonstrated markedly greater improvements compared to the control group. Pain scores (VAS) decreased by a mean difference of 2.56 (p < 0.001), functional independence (FIM) increased by 11.11 (p < 0.001), and self-efficacy improved by 8.71 (p < 0.001). Additionally, depressive symptoms (PHQ-9) reduced substantially with a mean difference of 6.15 (p < 0.001).

Conclusion: The CBSM provides an effective, structured strategy to enhance functional independence, self-efficacy, and mental well-being while reducing pain among individuals with orthopedic disabilities in rural settings.

## Introduction

A significant worldwide health concern is disability. It is described as any limitation or inability to carry out an activity in a way or within a range deemed typical for humans that arises from an organ's dysfunction. About 16% of the world's population lives with a major impairment at the moment. A rise in the prevalence of non-communicable diseases and population aging are contributing factors to this number's rise [1].

According to the social model of disability, social constraints, not an individual's limitations, are what cause their disabling character. Disability is a social construct that refers to the incapacity to perform one or more daily tasks on one's own at a "normal" level of functioning. In contrast, impairment is the "loss or abnormality of psychological, physiological, or anatomical structure or function" [2]. The functional, activity, and social interaction limitations, commonly associated with disability, are not quantified by years lived with disability [3]. Musculoskeletal disorders are pervasive, and they have a widespread effect. The precise number of people with this impairment in rural India is unknown. A locomotor impairment is an incapacity to do specific tasks related to moving oneself and objects from one location to another, and it is caused by a musculoskeletal or neurological condition [4].

Disability can significantly impact the quality of life and is compounded by additional medical, psychological, or environmental issues. Reducing disability through early rehabilitation can enhance community involvement and lessen its effects. Better health outcomes, greater access to educational institutions, increased economic engagement, lower poverty rates, and less reliance on the nondisabled population are all made possible by early disability detection and rehabilitation intervention [5]. A person's surroundings greatly impact the degree and experience of their disabilities. The full and effective engagement of people with disabilities in society on an equal basis with others is frequently impeded by inaccessible situations. Addressing these obstacles and assisting people with disabilities in their daily lives can lead to improvements in social engagement [1]. In contrast to a healthy society, which fosters positive attitudes toward people with disabilities and encourages social inclusion, negative views toward disabilities weaken people with disabilities and cause their social exclusion and isolation [6].

People frequently categorize individuals as belonging to either the in-group or the out-group. Those who are perceived as different, such as members of racial or sexual minorities, immigrants, or individuals with disabilities, are known as out-groups. Between the ages of 3 and 11, children's perspectives on disability change. While older kids get a wider perspective and become more inclusive, younger kids tend to concentrate on outward characteristics and may be less accepting of diversity. Even though implicit biases might endure in the absence of assistance, they can be lessened by exposure, education, and moral reasoning [6]. For children with disabilities, prompt identification and high-quality intervention are crucial for both individuals and society as a whole since they are expected to prevent and lessen the incidence and severity of chronic functional impairment [7].

Survivorship includes suggestions for the diagnosis, assessment, and management of physical and psychological issues brought on by impairment. It also presents a framework for care coordination as well as guidelines to promote healthy behaviors among survivors [8]. The survivorship model tackles the issues of trauma, and each trauma centre creates a specific outpatient program for trauma survivors, offering social support for patients together with patient-centred, high-quality physical and behavioural health care [9]. In remote locations, a survivorship model for people with disabilities improves access to community-based care, rehabilitation, and assistance. In addition to reducing travel, it fosters inclusion, tackles financial and emotional difficulties, and creates a more encouraging atmosphere [10]. Rather than aiming for a perfect "one-size-fits-all" survivorship care strategy, applying the best model under certain circumstances can improve outcomes and healthcare efficiency [11].

Social contacts have a big impact on the mental health and wellness of people with disabilities. To promote mental health and well-being, rehabilitation professionals should support people with disabilities and their significant others. This will help to ensure that healthy connections are formed and maintained and that there is enough support available. While integrating people with disabilities into social networks is a big task, it is equally important to improve the quality of their connections and to provide the type and degree of support that best suits their needs [12]. The structures and functions of the body for mobility are the special focus of three contributions. Given the increased physical limitations, relearning motor skills is necessary when faced with a physical impairment. This shows how shifting constraints lead to individual differences by contrasting the relearning principles in functional recovery and regular motor learning. Fitness and mobility are ways to engage with society [13]. The International Classification of Functioning, Disability and Health (ICF) and the previously mentioned functioning perspective were used to build the Prosthesis Orthosis Process model. Clinicians may guarantee that a patient-centred and holistic approach is applied throughout their clinical decision-making process by integrating ICF concepts into the model and promoting the usage of ICF codes and qualifiers. The four steps of the Prosthesis Orthosis Process model are assessment, goals, intervention, and outcome evaluation [14].

It will be necessary to address mobility and access difficulties more generally, in addition to health care, to meet the demands of disabled rural inhabitants. The survivorship model aims to alleviate the difficulties that people with orthopedic disabilities encounter in rural regions. It incorporates community-driven support networks, inclusive education, and easily available healthcare services to support physical and mental well-being. The concept empowers people to live satisfying lives by prioritizing early detection, customized rehabilitation, inclusive employment, and sustainable mobility solutions. Promoting equality of opportunity and lowering stigma through lobbying and education raises awareness and enhances the general standard of living for people living in underprivileged areas. Despite existing rehabilitation programs, there is no structured, community-driven survivorship model for rural orthopedic disability management. This study aims to bridge this gap. Thus, this study is an important step in comprehending the intricate dynamics of people with impairments. The study intends to provide important insights that can guide interventions, healthcare interventions, and practices targeted at enhancing the well-being of people with disabilities by looking at a variety of characteristics and their interactions [15].

Along with palliative care, illness prevention, treatment, and health promotion, rehabilitation is crucial to universal health coverage. In certain low- and middle-income nations, over half of the population does not receive the necessary rehabilitation services. Rehabilitation is a crucial component of universal health coverage and a major tactic for reaching the Sustainable Development Goal of ensuring healthy lives and promoting well-being for all at all ages. Numerous factors contribute to the ongoing unmet needs for rehabilitation around the world, such as a lack of national rehabilitation policies, plans, funding, and prioritization; a lack of rehabilitation services outside of urban areas, which results in lengthy waiting times; high out-of-pocket costs and non-existent or insufficient funding sources; and a lack of resources, such as assistive technology, equipment, and consumables [16]. The benefits of consolidating all the services that offer equipment for the disabled into a single entity are evident due to the broader range of duties and the understanding that overall services, both in the community and in the hospital, need to be improved [17,18]. It is becoming clearer that multiple morbidities affect functional impairment more than single morbidities, suggesting that different combinations of morbidities have a synergistic or additive influence on disability. When examining the disability burden among the elderly, it is crucial to consider multimorbidity due to the elevated risk of disability that is shown with the cumulative effect of multiple morbidities [19]. Public health strategies in India should consider this diversity because the burden of disability varies significantly between geographic regions and socioeconomic categories [20].

Compared to disability awareness, the school-based interventions were substantially more successful in enhancing attitudes toward disabilities. There is growing concern that many special needs children in inclusive settings experience social isolation despite the goal of integration being to foster healthy social connections between children with and without impairments. If we want to give these kids more chances for social success, we must evaluate programs that raise awareness of disabilities [21]. Parents' advocacy skills increased as a result of participating in facilitated parent groups. With more planned interventions, this capacity might be better supported over an extended time. Parent groups can provide solace to parents who live in under-resourced areas by allowing them to share their experiences, learn about services, and feel empowered [22]. Although results are less consistent than in the general population, social relationships are important for mental health and well-being in people with disabilities.

## Materials and methods

This study commenced after approval from the institutional protocol and ethics committee (protocol number: 138/2023-2024). The study’s primary goal was to evaluate the effect of the community-based survivorship model (CBSM) on individuals with orthopedic disabilities in rural areas. The comparative study was conducted with a total of 156 participants with orthopedic disabilities who were randomly selected for this study. The research was conducted in a community-based setup located in the rural area of Satara district, which was selected to evaluate the effectiveness of the survivorship model in a real-life community environment with limited access to rehabilitation services. The inclusion criteria comprised individuals aged between 30 and 55 years who had a locomotor impairment, fracture, or post-surgical musculoskeletal condition. Participants were excluded if they had a diagnosed tumour, severe psychiatric illness, or open wounds that could impede their safe participation in the prescribed rehabilitation activities. The patients were then randomly divided into two groups of 78 each. One group received a multimodal exercise program, while the other group received the integrated survivorship model.

Procedure

All patients were approached and given a detailed explanation of the study. Written and verbal informed consent was obtained from each patient. Demographic information was documented before initiating the study. Pre-assessment of the patients was conducted using data collection tools for both groups. The model was implemented for three consecutive three-month periods in one group, and the other group received a multimodal exercise program. Afterward, a post-assessment was conducted, and the results were interpreted.

This comparative study analyzes the challenges faced by disabled individuals in rural areas. The proposed model aimed to raise awareness about the availability of treatment and appropriate courses of action, ultimately improving the quality of life and physical health of these individuals. The study focused on disabled individuals living in rural areas. Approval was obtained from the protocol committee, and ethical clearance was obtained from the institutional ethical committee before the study. Subjects were selected based on inclusion and exclusion criteria. Based on the results of a previous study, a CBSM was developed.

Outcome measures

The outcome measures included the Visual Analogue Scale (VAS), Functional Independence Measure (FIM), Self-Efficacy Scale, and Patient Health Questionnaire-9 (PHQ-9) (Depression Score).

VAS

A subjective measure for pain intensity, ranging from 0 (no pain) to 10 (worst pain imaginable).

FIM

Assesses the level of functional independence in activities of daily living, covering motor and cognitive domains.

Self-Efficacy Scale

Evaluates an individual’s confidence in performing tasks and overcoming challenges, crucial for rehabilitation adherence.

PHQ-9

A standardized tool for measuring depression severity, with higher scores indicating greater depressive symptoms.

Intervention

Group A

Table 1 presents a phased rehabilitation approach designed to facilitate recovery, enhance functional capacity, and restore mobility following injury or disability. The protocol is divided into three distinct phases, each with specific goals, targeted exercises, and prescribed duration and repetitions to ensure progressive recovery.

**Table 1:** Multimodal exercise program TENS: transcutaneous electrical nerve stimulation; reps: repetitions

Phase	Goals	Exercises	Duration and Repetitions
Month 1: Acute phase (pain management and mobility restoration)	Reduce pain, control inflammation, prevent stiffness, and improve basic mobility	1. Pain management (cold/hot packs, TENS, ultrasound). 2. Passive range of motion (PROM). 3. Isometric strengthening (quadriceps, glutes, etc.). 4. Basic mobility training (bed mobility, transfers, weight shifting)	1. 10-15 min per session, 2-3 times/day. 2. 10 reps per joint, 2-3 times/day. 3. Hold for 5-10 sec, 10 reps, 2 times/day. 4. 15-20 min, 2 times/day
Month 2: Sub-acute phase (strength and endurance development)	Improve strength, stability, flexibility, and functional movement	1. Active-assisted and active ROM exercises. 2. Strengthening with resistance bands/light weights. 3. Proprioception and balance exercises (standing on one leg, wobble board, etc.). 4. Gait training (weight-bearing as tolerated, walking drills)	1. 15-20 min, 2-3 times/day. 2. 12-15 reps, 2-3 sets, 5 times/week. 3. Hold for 10 sec, 10 reps, 2 times/day. 4. 20-30 min, 5 times/week
Month 3: Advanced rehabilitation phase (functional and task-specific training)	Improve functional independence, enhance endurance, and restore normal movement patterns	1. Progressive strengthening (machines, free weights, bodyweight exercises). 2. Aerobic conditioning (cycling, walking, swimming). 3. Advanced balance and coordination drills. 4. Sport-specific/occupational training	1. 3-4 sets of 10-12 reps, 3-5 times/week. 2. 20-30 min, 4-5 times/week. 3. 10-15 min, 3 times/week. 4. 20-30 min, 2-3 times/week

Group B

**Figure 1:**
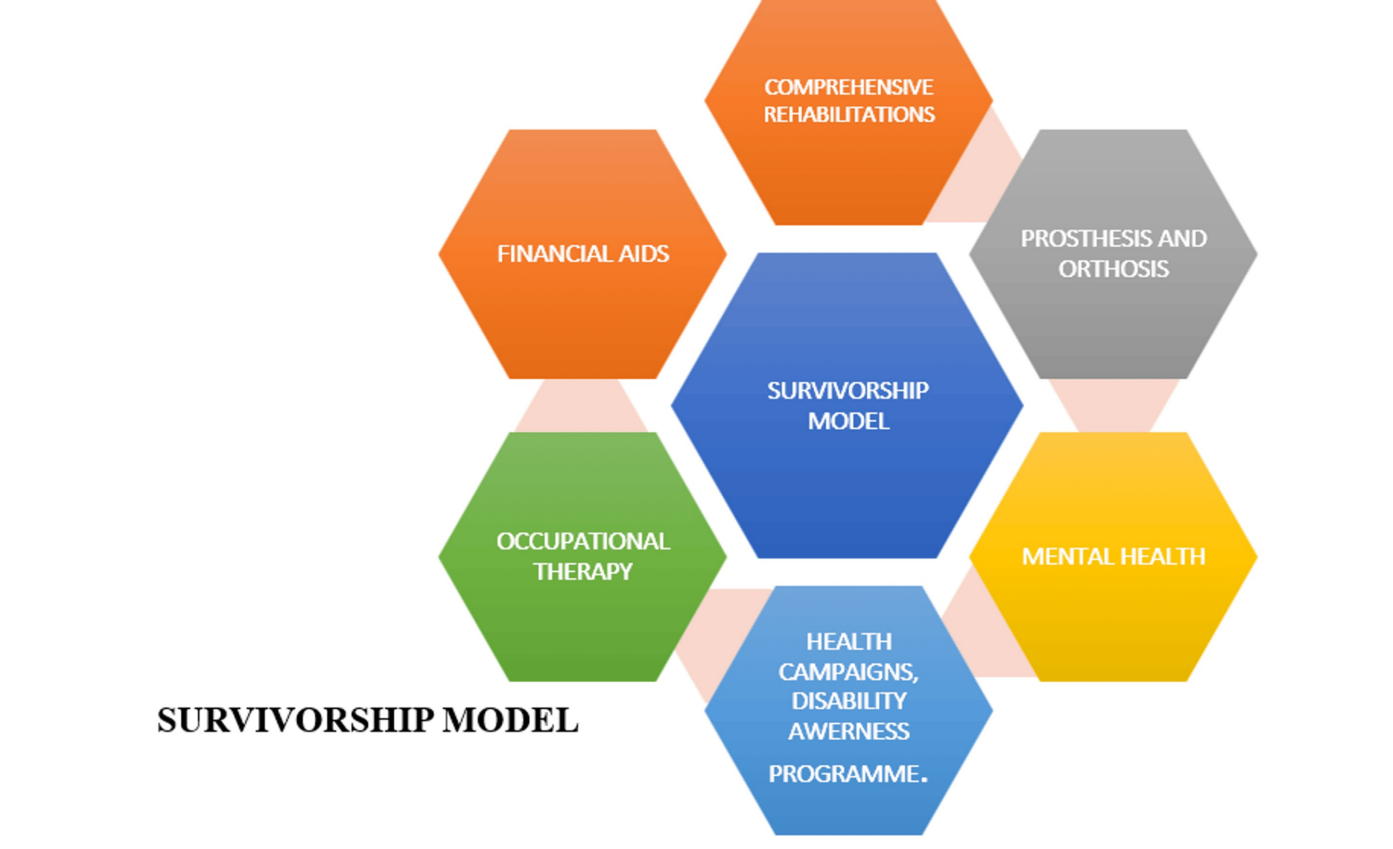
Survivorship model Credit: Image created by the authors.

The survivorship model comprises six fundamental components essential for the comprehensive rehabilitation and overall well-being of individuals, particularly those experiencing disabilities or injuries (Figure 1). This model adopts a multidisciplinary approach, integrating medical, psychological, social, and financial interventions to enhance functional recovery, promote independence, and improve quality of life.

Interpretation

The image depicts a survivorship model framework with interconnected components to improve the lives of individuals with orthopedic disabilities in rural areas. The model includes six key elements: (a) comprehensive rehabilitation; (b) prosthesis and orthosis; (c) mental health; (d) health campaigns and disability awareness programs; (e) occupational therapy; and (f) financial aid.

(a) Comprehensive Rehabilitation

Identify disability type: In rural areas, disability identification should be accomplished through community-based screening programs in which volunteers or trained health professionals examine people for indications of cognitive, sensory, and primarily physical impairments. Common disabilities can be identified through mass screening camps, and possible instances are identified using basic instruments like questionnaires, checklists, or observation techniques. Schools are essential in detecting youngsters' congenital abnormalities or developmental delays by conducting regular health examinations. Referrals for comprehensive exams, including physical examinations, musculoskeletal imaging, neurological evaluations, and vision or hearing tests, are made to local primary health centers (PHCs). Programs for early detection emphasize proactive monitoring during prenatal care visits or vaccination initiatives. This methodical technique guarantees the early identification of impairments, facilitating additional medical evaluation and action.

Orthopedic, neurological, and sensory/other: For efficient diagnosis, treatment, and resource allocation, especially in rural areas, disabilities must be divided into orthopedic, neurological, and sensory/other categories. This method expedites the identification process by associating particular infirmities with focused diagnostic instruments. Facilitating personalized interventions guarantees that people obtain customized therapies. Additionally, by guiding patients to the right specialists, categorization enhances referral systems, streamlines healthcare worker training, and maximizes resource utilization.

Pain management/medication: Pain management, medication, and assistive technology are crucial components of treatment, particularly in rural locations where access to healthcare may be restricted. A variety of non-pharmacological techniques, such as physical therapy and activities that promote mobility and lessen discomfort, can be used in pain management. Techniques like strengthening exercises and posture adjustment can also be useful for long-term pain alleviation. Medication may be required in certain situations to relieve extreme pain, but holistic methods are frequently still the major focus.

Advanced treatment/rehabilitation/specialized treatment: This involves treating conditions that result in impairment with medical techniques, tools, and treatments, such as surgical procedures like joint replacements or spinal operations for orthopedic problems. The goals of these therapies are to stop future health decline, alleviate symptoms, and restore function. The goal of rehabilitation is to assist people in regaining their independence and becoming more capable of carrying out daily duties. It is a protracted process that frequently entails close collaboration with a rehabilitation team to attain the optimum results. Individuals with certain disabilities or diseases will receive specialized care that is customized to meet their specific needs. With specialized treatment, patients are given guaranteed targeted care that optimizes their quality of life and chances of recovery while addressing their illness holistically.

Follow-up care and monitoring: By visiting patients, keeping an eye on their health, and educating families, community health workers (CHWs) can help close the gap. With personnel educated in disability care, local health clinics can offer routine examinations and referrals as necessary. People can obtain essential services by educating the community about the value of follow-up treatment and developing local mobility options. The support system can be further strengthened by working with non-governmental organizations (NGOs), which offer tools and resources for rehabilitation.

(b) Prosthesis and Orthosis

Mobile prosthetic clinics: These clinics can visit remote locations to offer orthosis, prosthetic fitting, rehabilitative care, and adjustments. With the right supplies and skilled staff, these clinics may make scheduled visits to villages or isolated communities, eliminating the need for people to travel great distances to urban areas.

Partnership with government programs and NGOs: Working with government health services, NGOs, and charity foundations can assist in lowering the cost of prosthetics and orthosis for those who require them. These groups provide financial assistance, discounted prosthetic devices, and assistance with transportation to care.

Training and capacity building: Local capacity to offer prosthetic and orthotic services can be increased by training healthcare professionals in the area, such as physiotherapists and prosthetists. People can receive care locally and reduce their need for outside assistance by setting up training programs in rural areas or making education more accessible in neighboring towns.

Subsidized transportation or local transportation networks: Access to care can be improved by offering subsidized or reasonably priced transportation to people who must travel to orthotic and prosthetic clinics. People with mobility disabilities can easily attend fitting and therapy appointments via local community transportation networks or government-supported initiatives.

(c) Mental Health

Cultural activities, inclusive sports, and awareness campaigns are community events that dispel stigma while promoting happiness and a sense of belonging. To provide a loving home environment for family members with disabilities, family therapy is essential because it gives caregivers the information and abilities they need to manage stress, support their emotional needs, and promote their independence. Individually designed skill development programs facilitate economic independence, increase self-esteem, and provide people with a sense of purpose through fulfilling employment. Critical services that might otherwise be unavailable are brought to patients' doorsteps by mobile mental health clinics, which provide on-site counseling, treatment, and psychiatric care. Through the establishment of a peer support network where members exchange experiences, work together to solve problems, and fight for their rights, self-help groups further empower people. By teaching parents, schools, and communities how to provide secure, caring environments and recognize the early warning signs of mental health issues, the approach starts with prevention. Through easily accessible counseling services, resilience-building initiatives, and techniques for emotional regulation, we will offer trauma-informed treatment to address problems such as abuse, neglect, or family upheavals. Peer support groups will be incorporated into individualized treatment programs that are created in collaboration with families to provide a comprehensive and individualized approach. We will use creative outlets like art and sports therapy, mindfulness exercises, and skill development programs to promote long-term growth and provide young people with a sense of purpose and self-worth. We will maintain their mental health development by providing continuity of treatment through integrated health, education, and social services, as well as frequent follow-ups.

(d) Health Campaigns and Disability Awareness Programs

Educating the public on the management, prevention, and treatment of orthopedic problems should be the main goal of health campaigns for people with orthopedic disabilities in rural areas. To increase public knowledge of common orthopedic problems such as joint pain, fractures, and musculoskeletal disorders, these programs can be carried out locally using CHWs, mobile health clinics, and village gatherings. Further issues can be avoided by encouraging physical therapy and activities that increase muscle strength and mobility. Improving independence requires educating people on how to use mobility aids, including wheelchairs, crutches, and orthotics. Additionally, the campaign can teach people how important it is to preserve bone health through exercise and a healthy diet.

In rural locations, a disability awareness program will greatly assist people with disabilities by creating a more welcoming and encouraging community. The initiative will reduce stigma and foster empathy by educating local leaders, families, and caregivers about the various kinds of disabilities and the skills of people with disabilities through workshops and seminars. This knowledge will improve integration in public areas, workplaces, and educational institutions, enabling people with disabilities to engage more fully in society. Additionally, the program will offer useful information on disability rights, mobility aids, and accessible infrastructure, making sure that people with disabilities have the resources and assistance they need to live freely.

(e) Occupational Therapy

In rural locations, occupational therapy is crucial for improving the quality of life for children, adolescents, and adults with orthopedic disabilities. An occupational therapist assists children as well as adolescents in creating adaptive strategies for everyday activities, including writing, dressing, and utilizing technology, all of which are essential for their education, independence, and social interaction. Occupational therapists for adults concentrate on enhancing functional independence in areas like employment, self-care, and housework. By using mobile clinics or community outreach, occupational therapists can reach people who might otherwise find it difficult to receive treatment in rural locations, where specialized care is frequently scarce. To ensure better long-term care, it also helps caregivers by teaching them practical techniques to help people with orthopedic disabilities at home.

(f) Financial Aid

People with orthopedic disabilities in rural locations can benefit from financial aid through many techniques. Through community leaders, local health facilities, and workshops that offer information on healthcare, rehabilitation, and assistive devices, government programs and subsidies should be promoted. Orthopedic treatment can be made cheap by partnering with local providers to offer health insurance programs that are subsidized or discounted. By providing financial aid, mobility aids, and advice in applying for government support, NGOs that prioritize disability rights can play a significant role. Fundraising initiatives or crowdfunding sites run by the community can also be used to pay for medical care or mobility assistance. To guarantee that assistance is easily accessible in rural areas, local healthcare providers are trained to help people find financial aid opportunities.

The participants allocated to Group B received a comprehensive, multi-component intervention based on the Survivorship Model framework, which integrates medical rehabilitation, psychosocial support, community engagement, and financial empowerment. This model was designed to address not only the physical aspects of musculoskeletal disability but also the broader determinants of health that influence long-term recovery and quality of life, to ensure replicability.

Table 2 shows the details of the goals, specific activities, and the recommended dosage and frequency for each phase of the program. The intervention was structured across three progressive phases: acute, sub-acute, and advanced.

**Table 2:** Survivorship model PROM: passive range of motion; NGO: non-government organization; ADLs: activities of daily living; OT: occupational therapy

Phase	Goals	Activities (Components)	Duration and Frequency
Month 1: Acute phase (pain management and holistic education)	Reduce pain, build awareness, and start self-management	- Comprehensive rehab: Pain management, PROM, isometrics, basic mobility. - Mental health: Individual counselling, family support, education. - Disability awareness: Basic sessions on rights, stigma, self-care	- Counselling: 30-45 min, 1/week. - Awareness: 30 min, 1/week
Month 2: Sub-acute phase (strengthening, peer support, prosthesis/orthosis)	Strength building, community integration, and assistive devices	- Comprehensive rehab: Active/active-assisted ROM, strengthening, balance training. - Prosthesis and orthosis: assessments, fitting clinics (if applicable). - Mental health: peer support groups. - Health campaigns: local community session	- Prosthesis: 1-2 hrs, once/month if needed. - Peer group: 1 hr, 2/week. - Community session: 1-2 hrs, once
Month 3: Advanced phase (functional training, community reintegration)	Improve functional independence, return to work, and financial linkages	- Occupational therapy: Task-specific ADL training, workplace adaptations. - Comprehensive rehab: Progressive strengthening, aerobic exercise. - Mental health: Ongoing counselling if needed. - Financial aids: Link to govt. programs, NGO support	- OT: 30-45 min, 2-3/week. - Rehab: same as Group A. - Counselling: as needed. - Financial aid: sessions with a social worker, 1-2 meetings

Statistical analyses

Both manual analysis and software analysis using IBM SPSS Statistics for Windows, Version 26 (Released 2020; IBM Corp., Armonk, New York, United States) were carried out. The numerical data were presented as means and standard deviations, and a paired t-test was employed to compare pre- and post-intervention data within the group. The between-group analysis was compared using an unpaired t-test. Continuous variables underwent normality testing. To assess significant changes over time, a repeated-measures analysis was conducted. A significance criterion of p < 0.0001 was set for all analyses to ensure robust statistical findings. This methodology provided a thorough evaluation of the data, integrating manual checks with advanced software analysis.

## Results

A total of 156 participants (Group A: control = 78, Group B: intervention = 78) were included in the study. The outcome measures analyzed were VAS, FIM, Self-Efficacy Scale, and PHQ-9.

Table 3 illustrates the demographic variability observed in this study, highlighting the characteristics of participants from both the multimodal exercise group and the survivorship model group. The demographic profile of participants indicated a balanced representation of genders across both groups, with a slight predominance of males overall. Age distribution was comparable between the control and experimental groups, with participants spread almost evenly across the 30-42 and 43-55 years age ranges. This suggests that both groups were demographically similar, minimizing the risk of confounding due to age or gender differences.

**Table 3:** Sociodemographic data of the participants

Demographic Variables	Total No. of Participants (N = 156)	Control Group (n = 78)	Experimental Group (n = 78)
Male	85 (54.5%)	42 (53.8%)	43 (55.1%)
Female	71 (45.5%)	36 (46.2%)	35 (44.9%)
Age (30-42 years)	79 (50.6%)	40 (51.3%)	39 (50.0%)
Age (43-55 years)	77 (49.4%)	38 (48.7%)	39 (50.0%)

Table 4 summarizes the within-group comparisons for all outcome measures, including effect sizes and confidence intervals. Overall, Group B demonstrated substantial improvements across pain (VAS), functional independence (FIM), self-efficacy, and PHQ-9. For instance, the reduction in pain for Group B showed a mean difference of 2.56 points (95% confidence interval (CI): 2.33 to 2.79; p < 0.001), indicating a large effect size in favour of the intervention. Similarly, functional independence improved by an average of 11.11 points (95% CI: 9.99 to 12.23; p < 0.001). Improvements in self-efficacy (mean difference: 8.71; 95% CI: 7.41 to 10.01; p < 0.001) and a significant reduction in PHQ-9 scores (mean difference: 6.15; 95% CI: 5.45 to 6.85; p < 0.001) further highlight the practical impact of the intervention. In contrast, Group A showed minimal or non-significant changes for most measures, with only a small improvement noted in self-efficacy.

**Table 4:** Within-group comparison: VAS, FIM, Self-Efficacy, and PHQ-9 VAS: Visual Analogue Scale; FIM: Functional Independence Measure; PHQ-9: Patient Health Questionnaire-9

Outcome Measure	Pre-intervention (Mean ± SD)	Post-intervention (Mean ± SD)	Mean Difference	95% CI	p-value	t-value
VAS (control group)	7.24 ± 1.10	5.49 ± 1.01	1.75	1.52-1.98	0.138	1.50
VAS (intervention group)	7.41 ± 0.95	4.85 ± 1.03	2.56	2.33-2.79	<0.001	3.31
FIM (control group)	85.61 ± 4.08	86.19 ± 4.99	0.58	0.54-1.70	0.478	0.71
FIM (intervention group)	84.39 ± 5.22	95.50 ± 4.94	11.11	9.99-12.23	<0.001	3.31
Self-Efficacy (control group)	39.61 ± 5.72	41.69 ± 6.30	2.08	0.67-3.49	0.040	2.08
Self-Efficacy (intervention group)	40.21 ± 6.43	48.92 ± 5.70	8.71	7.41-10.01	<0.001	3.31
PHQ-9 (control group)	11.79 ± 2.73	9.03 ± 2.60	2.76	2.18-3.34	0.558	0.59
PHQ-9 (intervention group)	12.85 ± 2.90	6.70 ± 3.07	6.15	5.45-6.85	<0.001	3.31

The post-intervention differences between the two groups are shown in Table 5. A statistically significant improvement was observed in the intervention group compared to the control group across all measures. The post-intervention comparisons between the two groups revealed significant differences. Group B had significantly lower VAS scores compared to Group A (p < 0.001). Similarly, FIM scores were higher in Group B (95.50 ± 4.94) compared to Group A (p < 0.001). Self-efficacy scores also improved more in Group B (48.92 ± 5.70) than in Group A (p < 0.001). Finally, PHQ-9 scores were significantly lower in Group B (6.70 ± 3.07) compared to Group A (p < 0.001), indicating better mental health outcomes. These results indicate that the intervention group had superior pain relief, functional independence, self-efficacy, and mental health, suggesting a more effective intervention.

**Table 5:** Between-group comparison of outcome measures using unpaired t-test VAS: Visual Analogue Scale; FIM: Functional Independence Measure; PHQ-9: Patient Health Questionnaire-9

Outcome Measure	Group A Post-intervention (Mean ± SD)	Group B Post-intervention (Mean ± SD)	p-value
VAS	5.49 ± 1.01	4.85 ± 1.03	<0.001
FIM	86.19 ± 4.99	95.50 ± 4.94	<0.001
Self-Efficacy	41.69 ± 6.30	48.92 ± 5.70	<0.001
PHQ-9	9.03 ± 2.60	6.70 ± 3.07	<0.001

## Discussion

The present study found that participants in the survivorship model group showed significant improvements in pain reduction (VAS scores), functional independence (FIM scores), self-efficacy, and reduced depression symptoms (PHQ-9 scores) compared to the control group. These findings demonstrate the effectiveness of the CBSM in enhancing physical and psychological outcomes for individuals with orthopedic disabilities in rural areas.

The study also emphasizes the potential of community-driven rehabilitation in improving functional outcomes, mobility, and quality of life. Tough et al. highlighted that rural residents with orthopedic disabilities come across many challenges, many of which are more severe than those faced by those living in urban areas. The immediate need for focused initiatives is highlighted by everyday challenges with mobility, accessibility, and employment. By implementing lifestyle and environmental changes, encouraging independence, and enhancing their quality of life, a survivorship model designed to meet their unique needs can help them deal with these problems. To close the awareness and care gap, it is essential to educate people and society about disabilities and therapeutic management. This work focuses on developing such a model using data from prior surveys and current disability research, with self-identified results directing its applicability and efficacy. Compared to people without impairments, people with disabilities utilize health care services more frequently, but they also experience major access disparities, such as lower coverage for general health care and other necessary services. It is challenging to compare results from different research because these discrepancies are exacerbated by differences in target populations, health outcomes, and access variables, such as gender and disability types. This emphasizes how urgently additional globally comparable statistics on health disparities, with an emphasis on service quality, affordability, and inclusivity, are needed. Making health care more inclusive can improve health outcomes for everyone, and addressing these persistent disparities is essential since they probably contribute to the life expectancy difference between individuals with and without disabilities [22].

Jarl and Ramstrand proposed the prosthetic and orthotic process (POP) model, integrating the ICF into prosthetics and orthotics practice. The model follows a cyclical approach with four key steps: assessment (medical history and physical examination), goal setting (targets at participation, activity, body function, and technical levels), intervention (evidence-based actions), and outcome evaluation (measuring effectiveness and patient satisfaction). This model enhances communication among rehabilitation professionals and promotes a holistic, patient-centered approach in clinical practice [14]. Mishra et al. emphasized that rehabilitation plays a fundamental role in achieving universal health coverage by facilitating independence and participation in daily activities. Unfortunately, many people in low- and middle-income nations do not have access due to a lack of resources, high expenses, and legislative gaps. It is essential to incorporate rehabilitation into primary care to address these issues and accomplish global health objectives [16].

Better care delivery can result from combining services for the disabled into one organization. Prosthetists and orthotists improve mobility, discomfort, and self-efficacy while restoring function by tailoring devices to each patient's needs. To lower the risk of disability and promote better health through preventive and appropriate nutrition, public health programs should concentrate on managing multimorbidity, particularly in the elderly, as multiple morbidities frequently exacerbate functional impairment [18,19].

Clinical implications

The survivorship model significantly enhances mobility, self-reliance, and overall functional independence in individuals with orthopedic disabilities. Integrating physical rehabilitation, prosthetic and orthotic support, mental health care, and financial assistance ensures a multidimensional approach to disability management. The model leverages local resources, reducing dependency on urban healthcare facilities and making rehabilitation more accessible to rural populations. Addressing psychological well-being alongside physical rehabilitation lowers depression scores (PHQ-9), improving the quality of life for individuals with disabilities.

Limitations

This study was conducted in a single rural area with a modest sample size, which may limit generalizability to broader populations. The follow-up duration was relatively short, restricting assessment of the long-term sustainability of the survivorship model’s effects. Future research should include larger, multicenter samples, longer follow-ups, and diverse settings to validate and refine this approach.

Future scope

Future models can incorporate tele-rehabilitation and AI-driven interventions to expand accessibility, especially in remote rural areas. Tailoring the model to children with congenital orthopedic disabilities and elderly individuals with mobility impairments can broaden its applicability. Collaboration with the government and NGOs can lead to the formal integration of survivorship models into public healthcare frameworks.

## Conclusions

The survivorship model provides a comprehensive and multidimensional framework to address the complex challenges faced by people with orthopedic disabilities living in rural areas. It incorporates essential components, including complete rehabilitation, the supply of orthotics and prostheses, mental health services, awareness campaigns, occupational therapy, and financial aid. Together, these elements seek to promote independence and improve quality of life by lowering obstacles to social interaction, healthcare, and mobility. This model's emphasis on early disability detection and classification guarantees prompt and focused therapies. Through the utilization of community-based resources such as schools, mobile clinics, and nearby health centers, the approach makes necessary services more accessible to people who might otherwise find them difficult to reach. By addressing both psychological well-being and functional independence, occupational therapy and mental health support are included, highlighting a person-centered approach. The approach also emphasizes how important it is to collaborate with NGOs, government initiatives, and community members to promote affordability, sustainability, and inclusivity. Transportation options, capacity-building programs, and financial aid systems all improve service accessibility, especially in rural and underserved areas. Significant progress may be made in promoting economic independence, enhancing health outcomes, and creating resilient communities where people with disabilities can flourish by implementing this model.
